# Mechanical Behavior of 3D Printed Poly(ethylene glycol) Diacrylate Hydrogels in Hydrated Conditions Investigated Using Atomic Force Microscopy

**DOI:** 10.1021/acsapm.3c00197

**Published:** 2023-04-05

**Authors:** Mohammad Hakim Khalili, Vishal Panchal, Alexander Dulebo, Sara Hawi, Rujing Zhang, Sandra Wilson, Eleftheria Dossi, Saurav Goel, Susan A. Impey, Adrianus Indrat Aria

**Affiliations:** † Surface Engineering and Precision Centre, School of Aerospace, Transport and Manufacturing, Cranfield University, Cranfield MK43 0AL, United Kingdom; ‡ Bruker UK Ltd., Banner Lane, Coventry CV4 9GH, United Kingdom; § Sophion Bioscience A/S, Baltorpvej 154, 2750 Ballerup, Denmark; ∥ Centre for Defence Chemistry, Cranfield University, Shrivenham, Swindon SN6 8LA, United Kingdom; ⊥ London South Bank University, 103 Borough Road, London SE1 0AA, United Kingdom; # University of Petroleum and Energy Studies, Dehradun 248007, India

**Keywords:** poly(ethylene glycol) diacrylate, 3D printing, AFM, heterogeneous modulus, cross-linking density, hydrogels

## Abstract

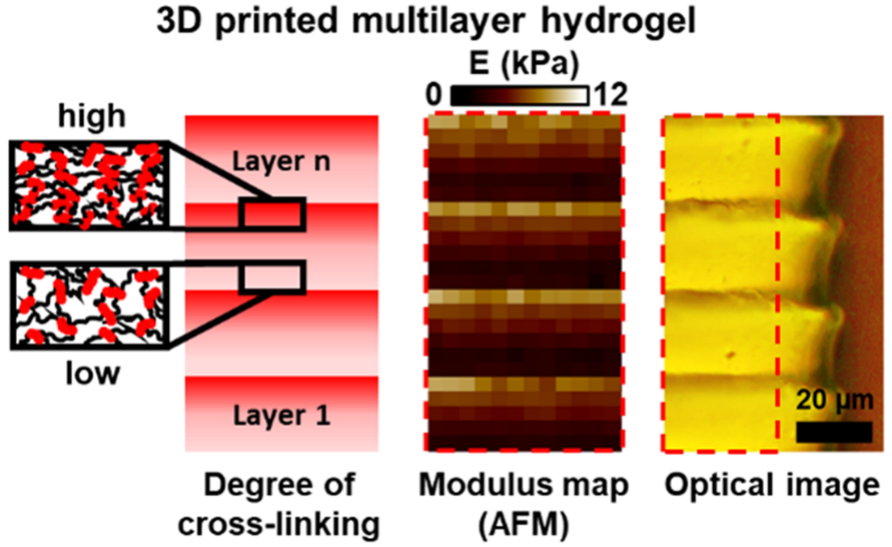

Three-dimensional (3D) printed hydrogels fabricated using light processing techniques are poised to replace conventional processing methods used in tissue engineering and organ-on-chip devices. An intrinsic potential problem remains related to structural heterogeneity translated in the degree of cross-linking of the printed layers. Poly(ethylene glycol) diacrylate (PEGDA) hydrogels were used to fabricate both 3D printed multilayer and control monolithic samples, which were then analyzed using atomic force microscopy (AFM) to assess their nanomechanical properties. The fabrication of the hydrogel samples involved layer-by-layer (LbL) projection lithography and bulk cross-linking processes. We evaluated the nanomechanical properties of both hydrogel types in a hydrated environment using the elastic modulus (*E*) as a measure to gain insight into their mechanical properties. We observed that *E* increases by 4-fold from 2.8 to 11.9 kPa transitioning from bottom to the top of a single printed layer in a multilayer sample. Such variations could not be seen in control monolithic sample. The variation within the printed layers is ascribed to heterogeneities caused by the photo-cross-linking process. This behavior was rationalized by spatial variation of the polymer cross-link density related to variations of light absorption within the layers attributed to spatial decay of light intensity during the photo-cross-linking process. More importantly, we observed a significant 44% increase in *E*, from 9.1 to 13.1 kPa, as the indentation advanced from the bottom to the top of the multilayer sample. This finding implies that mechanical heterogeneity is present throughout the entire structure, rather than being limited to each layer individually. These findings are critical for design, fabrication, and application engineers intending to use 3D printed multilayer PEGDA hydrogels for in vitro tissue engineering and organ-on-chip devices.

## 1 Introduction

Three-dimensional (3D) printed hydrogel photopolymers fabricated using light processing techniques such as digital light processing (DLP) and stereolithography (SLA) have been extensively used in tissue engineering and organ-on-chip devices.^1−3^ These photo-cross-linking techniques are fast, reproducible, and spatially precise for polymerization of liquid prepolymer solutions into multifunctional hydrogel biostructures with complex shapes.^4^ The fabrication involves layer-by-layer (LbL) cross-linking, in which the prepolymer solution is cross-linked by light irradiation, e.g., ultraviolet (UV), one layer at a time to allow a mold-free construction of a freestanding hydrogel structure.^5−7^ The photo-cross-linking process also allows property engineering of the biostructures through control of the fabrication parameters such as light dosage, step height, prepolymer molecular weight, and photoabsorber concentration.^8−11^

The surface properties of hydrogel biostructures on which the cells are cultured can have a strong influence on cell behaviors, including growth, migration, proliferation, differentiation, and tissue formation.^12−14^ For example, the development, organization, and differentiation of stem cells can be directed by engineering the surface elastic modulus (*E*), topography, and adhesion of hydrogel structures.^15−17^ In addition, the ability to measure contractile force of muscle tissue strips using 3D printed poly(ethylene glycol) diacrylate (PEGDA) hydrogel cantilevers requires precise engineering of *E* across the cantilever structure and selective tissue adhesion on the cantilever surface.^18^ Photo-cross-linked 3D PEGDA hydrogels produced using projection lithography allows for the spatial and temporal control of cross-linking and fabrication of various complex shapes.^19−21^

Despite the aforementioned advantages, previous studies have reported the existence of cross-linking gradients in photo-cross-linked 3D printed multilayer PEGDA hydrogels that result in inhomogeneous properties, e.g., *E*, across the printed layers.^22−25^ To date, atomic force microscopy (AFM) measurements were carried out in dried and cryosectioned biostructures;^26^ hence, there is a clear need for improving the fundamental understanding of outer surface properties of 3D printed multilayer hydrogels in realistic conditions. The research reported herein addresses this knowledge gap by systematically and thoroughly mapping the elastic behavior on the outer surface of 3D printed multilayer PEGDA hydrogels using AFM measurements in the hydrated state.^27−30^ While the findings presented herein are limited to elastic behavior, they provide a much-needed baseline understanding of the outer surface variation in *E* within the layer and across the entire structure that remained elusive prior to this study. This understanding is critical to allow tailoring of 3D printed hydrogel properties for the future design of tissue engineering scaffolds.

We performed AFM measurements on the outer surface of these PEGDA hydrogel samples when fully submerged in deionized (DI) water with a pH similar to a cell culture medium and in their swollen state.^31−36^ In this study, we found that hydrated multilayer sample with *M*_n_ of 700 g/mol had an *E* ranging from 2.8 to 11.9 kPa within a single layer. This variability was not observed in the monolithic control sample in which *E* remained at 32.3 ± 2.5 kPa within the same scale. A crucial finding from our study was that the *E* exhibited a substantial 44% increment, from 9.1 to 13.1 kPa, as the indentation progressed from the bottom to the top of the multilayer sample. This observation suggests that the presence of mechanical heterogeneity extends throughout the entire surface structure and is not confined to individual layers. It highlights the potential for tailoring the mechanical properties of 3D printed PEGDA by controlling the LbL printing process and the importance of considering cross-linking gradients in the design of tissue engineering scaffolds. This understanding provides insight into the surface modulus engineering of hydrogel biostructures critical for selective promotion or repulsion of biological tissues and can be extrapolated to other biopolymeric systems for the benefit of many tissue engineering and organ-on-chip applications.

## 2 Materials and Methods

### 2.1 Fabrication of PEGDA 3D Structures

**Figure 1:**
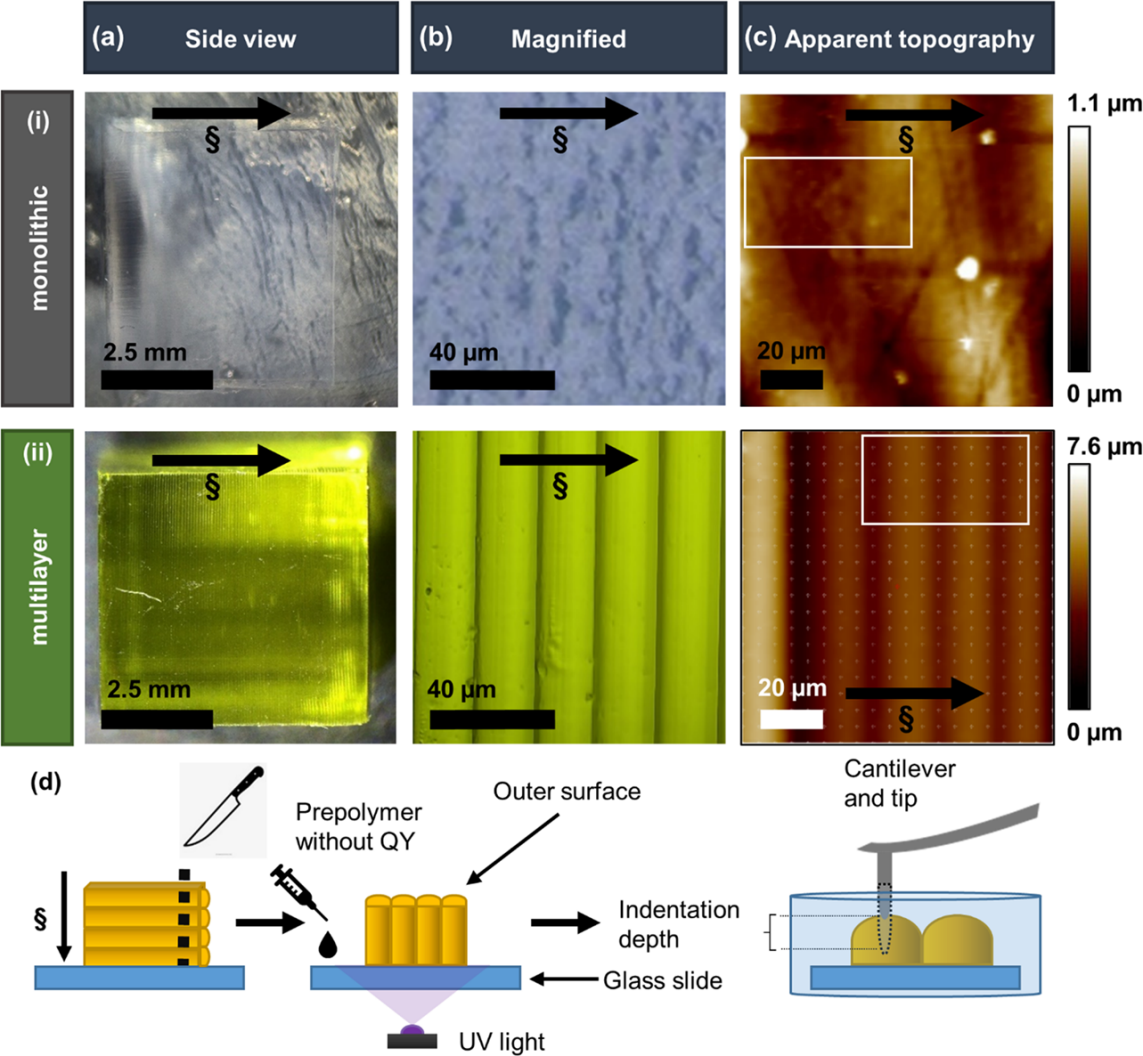
(a) Side view, (b) magnified optical microscopy images, and (c) apparent topography recorded with AFM peak force tapping mode (ramp) on the outer surface of (i) monolithic and (ii) multilayer PEGDA samples. (d) Schematic of sample preparation for the outer surface section of the multilayer sample. The white boxes were analyzed further for modulus measurements in Figure 2. Direction of projected light (§) is from top to bottom of the structure.

3D printed multilayer PEGDA sample was synthesized from PEGDA monomer (*M*_n_, 700 g/mol) dissolved in DI water (DIW, resistivity of 18.2 MΩ·cm) at a weight concentration of 20 wt %. The prepolymer PEGDA solution was prepared by mixing with 5 mg/mL photoinitiator lithium phenyl-2,4,6-trimethylbenzoylphosphinate (LAP, ≥95%) and 9 mg/mL photoabsorber Quinoline Yellow (QY). Due to their excellent water solubility and cytocompatibility, LAP and QY were selected for this study.^37^ The light source was a UV light emitting diode (LED) with a wavelength of 365 nm at an intensity of 20 mW/cm^2^. For multilayer sample, each layer (composed of 250 layers, Figure 1aii) was exposed to 3 s of UV light to achieve cross-linking (Figure S1a). Here, the top section of a printed layer refers to the area closer to the light source, while the bottom section refers to the farther one. As the 3D structure is printed LbL from bottom to top, the bottommost layer refers to the first printed layer, while the topmost layer refers to the latest printed one. Monolithic sample (Figure 1ai) was fabricated as control sample by pouring a prepolymer solution of PEGDA and LAP without QY photoabsorber into a silicone mold with 5 mm structural height. The sample was then exposed to a UV light source from the top with the same intensity of 20 mW/cm^2^ for 3 s (Figure S1b). The detailed protocols for preparing the samples can be found in our previous study.^38^

### 2.2 Sample Preparation for AFM Imaging

Both types of fabricated PEGDA hydrogel samples were cut vertically across the printed layers to prepare outer surface sections (Figure 1d) approximately 3 mm thick using a 100 μm thick stainless steel blade. A droplet of the prepolymer solution without QY was applied on an acrylate treated glass slide. The cut section of the sample was then placed on the glass slide and exposed to a UV light from the bottom, so it adhered to the glass slide (Figure 1d). The prepared samples attached to the glass slide were transferred to a 60 mm Petri dish and fixed to the bottom of the dish using epoxy superglue. After 1 min, DIW was added to ensure that the samples remain hydrated. The samples were stored for 24 h at a room temperature of 20–22 °C before AFM measurements to ensure the samples were at equilibrium. The water level was kept just above the sample height to reduce the risk of damage to the AFM head. Measurements were performed in DIW at controlled temperature (21 °C) and relative humidity (35–40%).

### 2.3 AFM Quantitative Imaging

AFM measurements were obtained using a Bruker Dimension Icon AFM. Data analysis and image processing were conducted with NanoScope Analysis software (version 1.85) and JPK SPM Data Processing 8.0.13. A Nature protocol for measuring *E* of soft culture tissue and 3D hydrogels using AFM was followed.^34^ A precalibrated BioAFM spherical tip of radius of 3.46 μm and low spring constant of 0.217 N/m was selected to induce sufficient deformation without damaging the sample while retaining their sensitivity.^39^ Deflection sensitivity of 28.69 nm/V was measured by multiple indents into a silicone sample and analyzing the force–distance curves. A droplet of DIW was dropped gently on the tip of the AFM probe, creating a water dome to integrate with the top surface of the hydrogel and the surrounding water system when contacting the sample. A scanning area of 90 μm × 90 μm was measured at a predetermined location within the sample 2.5 mm from its bottom right corner and at a height of 2.5 mm from the bottom of the sample. A grid of 20 × 20 (total of 400 indents) within the 90 × 90 μm scanning area was set with spacing between each indent set to 4.737 μm (Figure S3).

The indentations were carried out in contact mode with a maximum load of 60 nN. The raster scan was adjusted with the tip scanned over the sample surface at speeds of 30 and 26 μm/s and sample rate of 3,125 and 3,333 Hz for multilayer and monolithic samples, respectively. The *z*-length scale was fixed at 5 and 4 μm for multilayer and monolithic samples, respectively. The load and unload profiles of 164 and 154 ms for multilayer and monolithic samples, respectively, were developed.

To measure the apparent topography of the sample surface, initial scans were performed in ramp mode where the cantilever was moved rapidly along the *x*-axis and slowly along the *y*-axis while maintaining a constant force of 60 nN. *E* was calculated based on the average of 6 indent points across the *x*-axis. The change in *E* across the height of the multilayer sample was measured at 1.25, 2.50, and 3.00 mm from the bottom of the sample using a single line function of 90 μm with indent spacing of 1 μm. The measurements were taken at the peak section of each layer with an average *E* of 3 indents at the middle of the layer. Details on the fitting methods of Hertz and Oliver and Phar can be found in the Supporting Information.

## 3 Results

Figure 1c shows the apparent topography for the outer surface of both monolithic and multilayer PEGDA samples. The color code, from dark brown to white, indicates the apparent height variations within each sample from the lowest to the highest point, respectively. Figure 1cii shows the outer surface topography of the multilayer sample where the dark vertical lines are indicative of the interface of each printed layer and the lighter areas are indicative of the printed layers themselves. Such topography is not visible in the monolithic sample, which was produced using bulk cross-linking (Figure 1ci).

**Figure 2:**
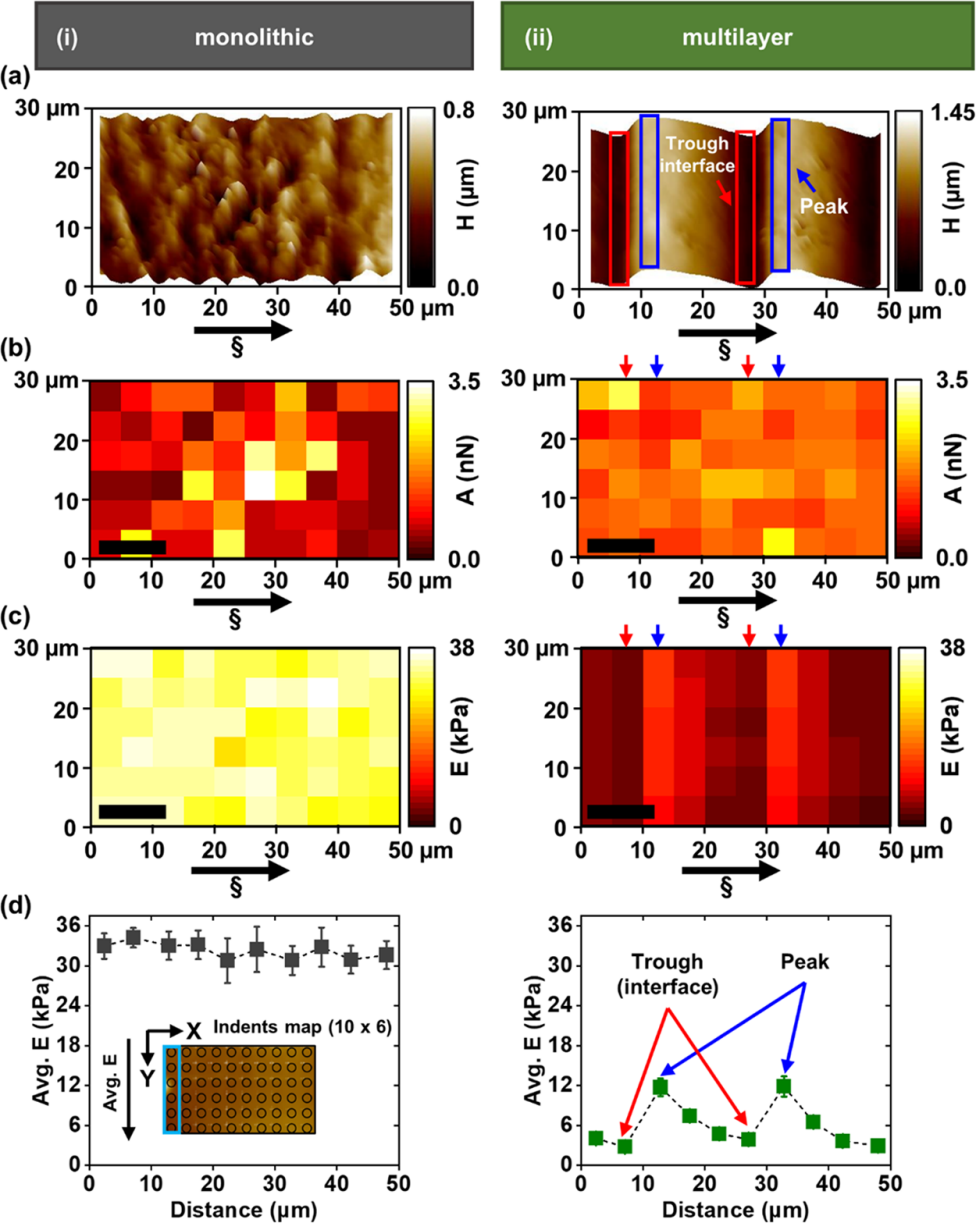
For (i) monolithic and (ii) multilayer PEGDA samples, (a) AFM ramp images of the surface topography with indentation map 50 × 30 μm. The red and blue boxes indicate the peak and trough (interface) positions on the multilayer sample. (b) Adhesion map of 10 × 6 indents with row and column spacing of 4.74 μm, indicating the adhesion force between the sample and AFM tip. (c) *E* map showing variations in modulus in both samples. (d) *E* calculated by averaging 6 indents at each point across the *x*-axis (Figure S2b). Error bars represent standard deviation from a mean of *n* = 6. Some error bars are not visible as they are smaller than the data point symbols. All dashed lines are only guides for viewing. Direction of projected light (§) is from top to bottom of the structure. *H*, *A*, and *E* stand for height, adhesion, and elastic modulus, respectively. Scale bars in all graphs are approximately 10 μm.

Looking at Figure 2ai, random height variations in monolithic sample are visible, which suggests that the sample surface is not smooth. In Figure 2aii, the height variations seem to be of a repeated pattern across the multilayer sample from left to right indicating the outer surface of the printed layers across the sample height. The darker areas, red boxes, are indicative of the shallow section of the sample surface close to the interfaces between two consecutive printed layers (trough), and the bright areas, blue boxes, are showing the raised areas on the sample surface (peak). Adhesion maps (Figure 2bi,bii) show the adhesion force between the scanning tip and the surface of both samples is less than 3.5 nN, which is relatively low and insignificant in comparison with the maximum applied load of 60 nN.

The results also suggest that the surface topography does not influence the measured adhesion force (Figure 2bi,bii). Figure 2ci shows *E* for monolithic sample ranges from 26 to 37 kPa. As the sample is traversed through each layer, *E* gradually increases from right to left, displaying a periodic pattern in the multilayer sample (Figure 2cii). The results obtained using the Hertz model (Figure 2ci,cii) in comparison with an upper fitting range (Figure S4bi,bii), as well as the Oliver and Phar method (Figure S4ci,cii), show a similar overall trend and are comparable. Average *E* increases by 4-fold from 2.8 to 11.9 kPa when comparing the bottom and the top section of the same layer in the multilayer sample (Figure 2dii). Such gradual variations were not visible on the calculated average *E* in the monolithic sample within the scanning area (Figure 2di). The results shown in (Figure 2di,dii) indicate that the monolithic sample exhibits twice the value of *E* compared to the multilayer sample.

**Figure 3:**
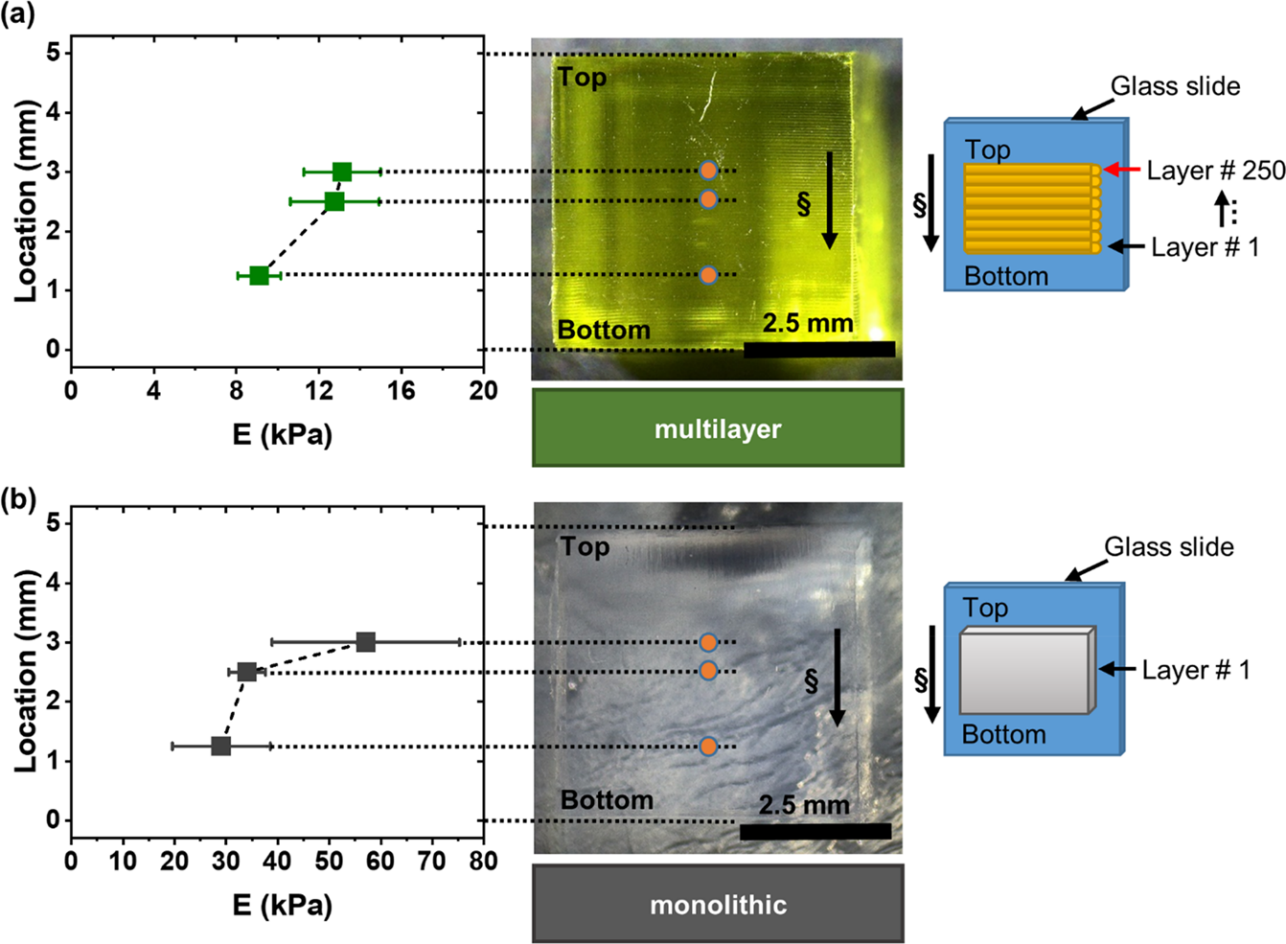
Change in *E* measured at three different locations across the height on the outer surface of the (a) multilayer and (b) monolithic PEGDA samples. *E* calculated (a) by the average of 3 indents on 3 layers at each height and (b) by the average of 90 indents at each height (Figure S5). Error bars in (a) represent combined uncertainty of *n* = 3 with 3 indents for each *n* and (b) represent standard deviation from a mean for *n* = 90. Direction of projected light (§) is from top to bottom of the structure. All dashed lines are for ease of viewing.

In the multilayer sample, *E* increases by 44% across the height of the structure, going from 9.1 to 13.1 kPa when moving upward from 1.25 to 3.00 mm at the bottom of the structure (Figure 3a). Conversely, the monolithic sample displays an increase in *E* of 120%, going from 29 to 57 kPa when moving from 1.25 to 3.00 mm at the bottom of the structure (Figure 3b). The data obtained from the monolithic sample reveal that the measured *E* values were notably higher than those of the multilayer sample, ranging between 2.6 and 4.5 times higher (Figure 3a,b). In particular, at a height of 3 mm, the average *E* for the monolithic sample was 57 kPa, compared to only 13.1 kPa for the multilayer sample.

## 4 Discussions

**Figure 4:**
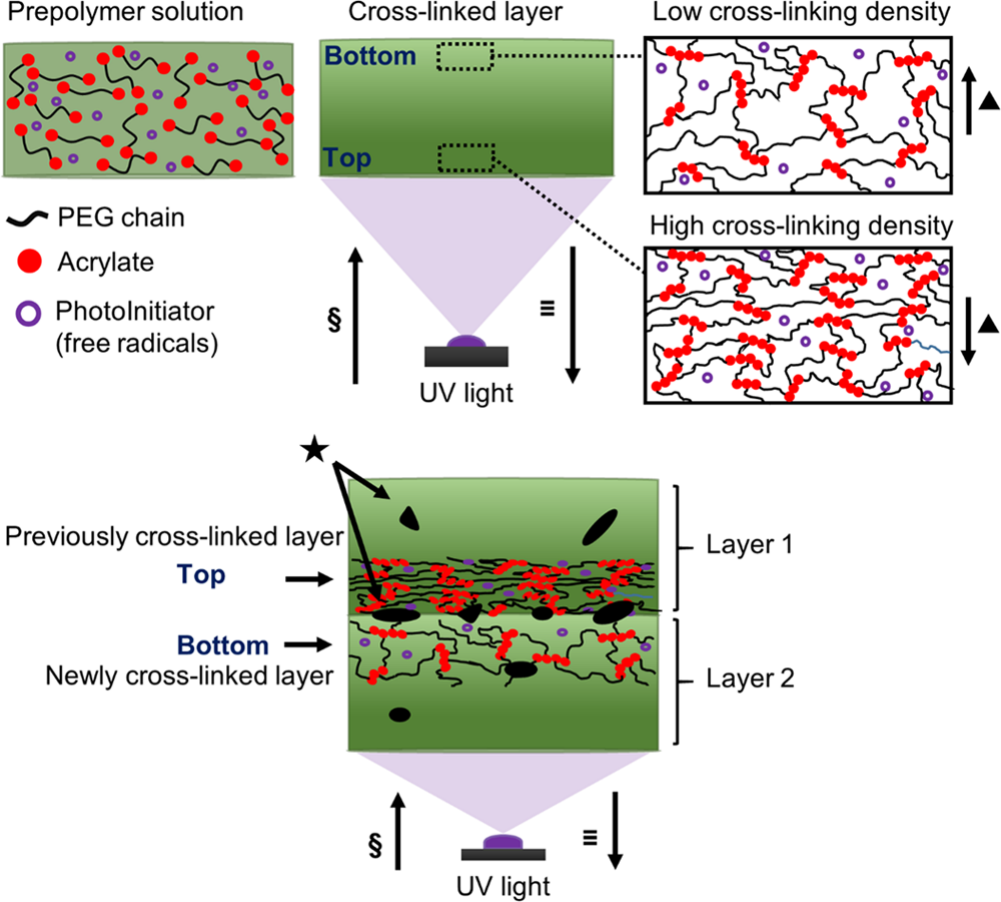
Schematic of the prepolymer solution (top left), single cross-linked layer (top middle), two different cross-linking densities at the top and bottom of each layer (top right),^49^ and two consecutive cross-linked layers with different amounts of cross-linking at the interface (bottom middle). §: Projected light direction; ≡: printing direction; ▲: amount of available residual free radicals at the top and bottom of a cross-linked layer; ★: empty pockets of voids swollen due to water uptake. Adapted by the authors from Figure 2, p6^49^ licensed under CC BY.

The peaks and troughs observed in the surface topography of the multilayer PEGDA sample are inherent parts of the structure and reflect how the structure is printed using projection lithography. AFM measurements were not carried out on the cross-sectioned multilayer sample, as mechanical cutting using thin blades resulted in the introduction of unnecessary cutting artifact to the surface topography (Figures S6 and S7). As the multilayer sample structures are intended for use in tissue engineering studies, where they will be in direct contact with biological cells in their hydrated state, it is best to investigate their outer surface nanomechanical behavior in a similar state and original surface topography to ensure the results are more representative of the actual model. While cell study is outside the scope of this current investigation, the understanding of the nanomechanical behavior will help elucidate the cell adhesion, integration, and tissue attachment onto the 3D printed structures.^13,16,18^ This will also help to ensure the measured *E* is not an overestimation of the real value when compared with samples measured in their dry state.^26,28,30^ The gradient in *E* observed in each layer of the multilayer sample can be understood by gaining a deeper insight into the printing process. As projection lithography is a bottom-up printing approach, during the process, light penetration depth governs the kinetics of cross-linking which in turn is controlled by the concentration of the photoabsorber and photoinitiator in the prepolymer solution (Figure S10).^38^ Choosing the right photoabsorber and photoinitiator is crucial for achieving optimal functionality, printing fidelity, and cross-linking rate in biomedical applications. In addition to the low risk of cytotoxicity, key factors to consider when selecting a photoabsorber and photoinitiator include their absorption spectrum, molar extinction coefficient, and water solubility.^40^ Methyl orange and diaminobenzophenone were not practical for use because they were poorly soluble in water, despite their initial consideration for their stronger absorption at 365 nm.^41^ Other photoabsorbers such as 2-hydroxy-4-methoxy-benzophenone-5-sulfonic acid (HMBS) were reported to be cytotoxic.^42^ However, QY photoabsorber, which is a water-soluble commercial food dye, shows low cytotoxicity and a good absorbance at 365 nm.^41^ For selection of photoinitiator, LAP’s low cytotoxicity has already been established, and multiple studies in the area of tissue engineering and cell seeding have used it successfully.^42^ Moreover, LAP is highly water-soluble with high molar absorptivity (ε ≈ 200 M^–1^ cm^–1^) and has been successfully used in DLP and SLA based printing.^43,44^ Alternative photoinitiators like Irgacure-2959, Eosin Y, and (2,2,6,6-tetramethylpiperidin-1-yl)oxyl (TEMPO) were found to be inadequate due to factors such as low solubility in water, insufficient absorption at a wavelength of 365 nm (absorbance range from 400 to 800 nm), and cytotoxicity, respectively (see Supporting Information Section 10).^40,42^ Since UV light attenuates and decreases in intensity when it propagates through a photoabsorber containing solution, the control over photoabsorber concentration to limit the penetration of UV light into the prepolymer solution enables control over the thickness of the cross-linked layer.^45^ This phenomenon causes an exposure gradient through the layer, resulting in higher cross-linking at the top section of each layer closest to the UV light. The bottom of the layer may not even reach the full gelation point (Figure 4).^46^

It is critical to find the cross-linking depth, as it signifies the point at which the light intensity is inadequate to cause cross-linking that results in the prepolymer solution remaining in a liquid state. Here, the extinction coefficient of QY photoabsorber is approximated using Beer–Lambert law and then used to identify the optimal layer thickness (Figure S8).^45^ Previous investigation suggested that the optimal concentrations for QY photoabsorber and LAP were 9 and 5 mg/mL, respectively, for the production of open channel structures.^37^ For the same concentrations, we measured the cross-linking depth to be 46 ± 2 μm.^38^ Based on this information, the layer thickness was set at approximately half of the cross-linking depth, or 20 μm, assuming that each layer would undergo two exposures during the LbL printing process to enhance layer–layer adhesion. However, upon closer examination, it was discovered that, during the printing process, only a small portion of the upper layer, closer to the interface of the previously formed layer, undergoes double exposure (Figures S9 and S10). This results in the creation of a highly cross-linked planar sublayer and may also cause additional volume within the structure, leading to issues with dimensional accuracy.^47^ This is likely the cause of the hump-shaped feature present at the top of each layer before every interface in the printed structure (Figure S11). The exposure gradient through the layer and the double exposure of the top section of each layer results in a gradient cross-linking density across each printed layer. This exacerbates the gradient of cross-linking degree and adversely affects the mechanical properties across the thickness of the layer, which agrees with the results in Figure 2.^24^

In our previous study, we found that the monolithic sample, which did not contain the QY photoabsorber in its prepolymer solution, had the highest degree of cross-linking and highest *E*, as indicated by both ^1^H NMR spectra and nanoindentation measurements. On the other hand, the multilayer sample, which had the highest concentration of QY photoabsorber, exhibited a lower degree of cross-linking as well as *E*. This observation suggests that a higher concentration of QY photoabsorber in the prepolymer solution leads to a reduction in the degree of cross-linking in the cross-linked hydrogel, as shown in Figures 2id,iid, and 3a,b.^48^ We have also showed the effect of cross-linking on water and sol–gel content in the monolithic and multilayer samples with equilibrium water content of 72.6% and 75%, respectively.^38^ Gel fractions were measured to be 27.4% and 25%, while sol percentages were 2.5% and 5.9% for the monolithic and multilayer samples, respectively.^38^

**Figure 5:**
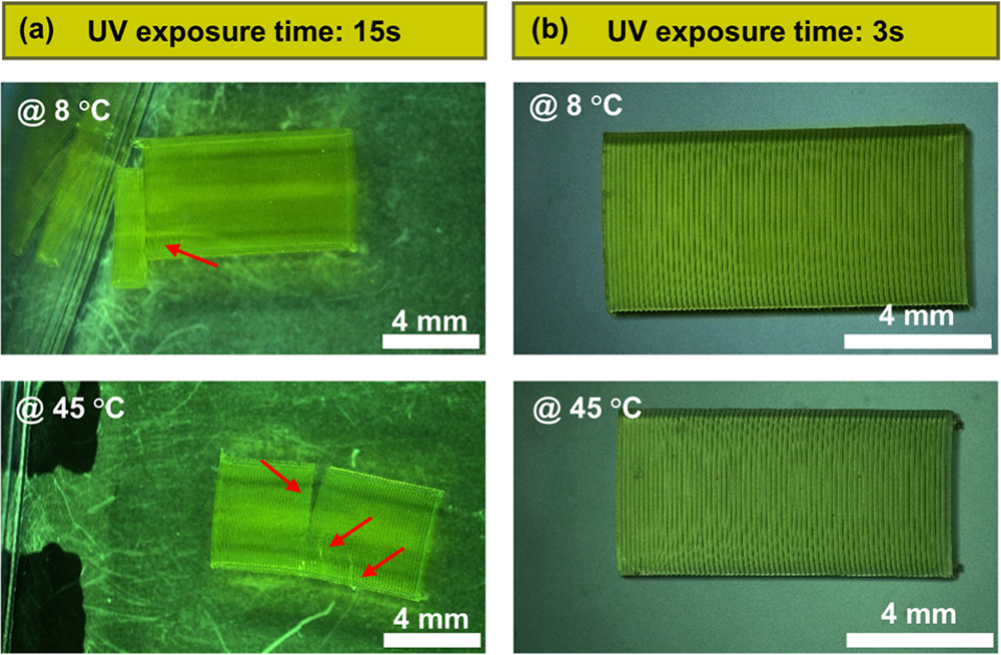
Optical microscope images of multilayer PEGDA samples exposed to (a) 15 s and (b) 3 s of UV light stored at 8 and 45 °C. Layer delamination and cracking is observed in samples exposed to 15 s of UV light.

As described above, the lowest *E* is recorded at a trough (Figure 2dii), which is the interface between layers. The observed tearing suggests that the interface between the layers is indeed the weakest point across the entire structure due to its highest heterogeneity in cross-linking density. This agrees with our previous study that suggests, due to the nature of the LbL cross-linking process, there are inherent structural imperfections in projection based printing processes.^48^ For each layer upon cross-linking, localized areas of unreacted monomer are formed which affect the stiffness of the material at the interface. These pockets of voids or defects could result in variations in the defect density and lower *E* at the interface of the multilayer sample.^48^ NMR characterization carried out in our studies suggests that the presence of localized unreacted prepolymer regions is mainly caused by the insufficiency of the UV light dose of 120 mJ/cm^2^ to fully cross-link the exposed prepolymer solution in the presence of the QY photoabsorber with a concentration of 9 mg/mL.^48^ Traces of unreacted PEGDA’s absorption, which corresponds to the protons of −CH_2_CH_2_O– and CH_2_=CH— groups, could be clearly observed in the NMR spectra in the multilayer sample.^38,48^ A higher UV dosage may help reduce the possibility of these localized regions within the layers. However, exposing the layers to a higher dosage of UV light, e.g., 15 s, which is equivalent to 600 mJ/cm^2^, may lead to overexposure. This increases the risk of tearing into individual layers and cracking within the hydrogel structure, which is observed when multilayer samples are stored at 8 and 45 °C in their hydrated state (Figure 5a).

Delamination at the interface of the multilayer sample is attributed to the lower adhesion yield stress than the cohesive yield stress, meaning layer–layer adhesion is not necessarily 100% chemical in nature.^45^ The cross-linking process occurs through free radical transfer where the LAP photoinitiator absorbs UV light to form reactive species and initiate reaction cascades, by which the PEGDA monomers react to form a cross-linked polymer.^12^ The cross-linking process terminates through coupling or disproportionation when free radicals become topologically constrained and cannot form links with the unreacted monomers and oligomers remaining in the previously cross-linked layer.^45^ Therefore, the deficiency in free radicals and/or their inability to diffuse in the previously cross-linked layer and their failure to react with the unreacted polymer and oligomers within the layer results in an inadequate cross-linking at the interface, which in turns results in weak adhesion between two consecutive cross-linked layers (Figure 4). There is also the possibility of bubbles within the prepolymer solution that may appear on the surface of the vat and under the exposed area while the printing is in process, which may result in the appearance of the voids within the multilayer sample especially at the interface. It is necessary to verify that the various layers are completely bonded by covalent cross-linking throughout the printing process. The examination of covalent cross-linking exclusively at the interface using NMR is, however, incredibly challenging due to its relatively low spatial resolution. Nonetheless, our prior research has demonstrated a definitive correlation between cross-linking and mechanical properties. Thus, we employed AFM to verify the existence of cross-linking and measure the spatial variation across the layers. Note that the observed reduction in modulus could also be attributed to the presence of voids or defects, suggesting that the layers may not be fully covalently cross-linked at the interfaces.^48^ It is crucial to investigate and find the optimum amount of LAP photoinitiator, QY photoabsorber, and UV dosage required to minimize the chance of the appearance of these pockets of voids and increase the adhesion at the interfaces of the cross-linked layers.

In addition to a gradient in *E* within each layer, the printed multilayer sample also exhibits a gradient in *E* from the top layer to bottom layer of the structure (Figure 3a,b). This strongly suggests that the mechanical properties of the multilayer sample are heterogeneous across the entire height of the structure. Nonetheless, the measured gradient in *E* across the height of the monolithic sample is significantly higher than that of the multilayer sample. This can be explained based on the curing reaction that occurs in the photo-cross-linking processes. Initially, when the UV light is directed from the top of the prepolymer solution, gelation occurs when the degree of cross-linking is enough to guarantee the formation of a solid structure that can result in soluble or insoluble common solvents, which has a low degree of cross-linking. This results in the coexistence of both gel and sol phases. The curing reaction continues with the same mechanism, and the amount of sol phase in the system decreases until completion and formation of an insoluble hydrogel which is highly cross-linked.^51^ From this point, the reaction becomes very slowly controlled by the diffusion of the reactive species. It takes a much longer time for the reactive groups to find each other and form links as the bulk density of cross-linking becomes higher. This diffusion-controlled effect may lead to the formation of nonhomogeneous hydrogel structures with heterogeneous mechanical properties.^51^

In the case of a monolithic sample, where the layer thickness is 5 mm, the corresponding diffusion time is considerably higher than that of the multilayer. In multilayer sample, where the layer thickness is 20 μm, diffusion of active groups from the top to bottom of the structure is highly unlikely. The vertical variation in *E* is stronger in monolithic sample compared to multilayer sample due to the fact that, in monolithic sample, initiated species at the top of each layer require more time to diffuse to the bottom, causing a stronger variation.

The findings presented herein provide a baseline understanding of spatial variation in the elastic behavior of 3D multilayer PEGDA within each layer, at the layer interface, and across the entire height of the structures. One important aspect that has yet to be investigated is the viscoelastic behavior, including hysteresis, creep, and storage/loss moduli of 3D printed PEGDA hydrogels. The approach used in this study can be extrapolated to systematically investigate the effect of LbL synthesis parameters on spatiotemporal variation in mechanical properties to enable tailoring of the local and global mechanical properties of hydrogel structures. Future studies may benefit from more advanced SPM techniques such as nano-DMA to fully elucidate the time dependent behavior of hydrogels in physiologically relevant environments.

## 5 Conclusions

In our study, we successfully mapped the apparent elastic modulus of the 3D printed multilayer PEGDA hydrogel sample and compared them to the monolithic PEGDA hydrogel as a control sample in their native hydrated state. It was observed that a gradient in the elastic modulus in the printed layers of a multilayer sample increases moving upward from the bottom to the top of individual layers. *E* of a multilayer sample at the middle of the printed structure increases 4-fold, from 2.8 to 11.9 kPa, when moving from the bottom to the top of each layer. Such variations were not observed in the monolithic sample. Experiments revealed that the LbL UV cross-linking process using projection lithography results in a cross-linking density gradient within each layer due to the spatial decay of the light intensity as the light propagates throughout the prepolymer, printing solution.

More importantly, the investigation at 3 different spatial locations across the height of both monolithic and multilayer samples shows a considerable difference in *E* from the bottom to the top of the structure. Even though the variations in *E* in the monolithic sample were significantly higher than that of the multilayer, an increase of 44% in *E* for a multilayer sample across its height from 9.1 to 13.1 kPa is considered large. Reducing the layer thickness in the multilayer sample and postprinting UV curing may help in improving the overall surface mechanical homogeneity of the 3D printed PEGDA structures. These findings can significantly help in understanding how biological cells and tissues interact when in contact with the surface of a heterogeneous material with mechanical property gradients and how this feature can be utilized to guide cell integration, attachment, and tissue formation for different biological applications.

## Data Availability

Data underlying this study can be accessed through CORD at 10.17862/cranfield.rd.19208487.

## Supporting Information Available

Schematic of the printing setup, indentation maps, images of indentation locations on samples, explanations of contact point identification, explanation and schematic of surface topography on measurements, representative force–distance curves, comparison of elastic modulus maps, explanation on measurement of cross-linking depth, optical images of outer surface topography, and schematic of exposed areas inside a vat (PDF)
