# Supplementary material for: Mechanical Behavior of 3D Printed Poly(ethylene glycol) Diacrylate Hydrogels in Hydrated Conditions Investigated Using Atomic Force Microscopy
Source: ACS Appl Polym Mater. 2023 Apr 5;5(4):3034–42. doi: 10.1021/acsapm.3c00197 (PMC10111335; doi:10.1021/acsapm.3c00197)
Supplement: Supplementary file 1 — ap3c00197_si_001.pdf [file ap3c00197_si_001.pdf]

**SUPPORTING INFORMATION**

# "Mechanical Behavior of 3D Printed Poly(ethylene glycol) Diacrylate Hydrogels in Hydrated Conditions Investigated Using Atomic Force Microscopy "

***Mohammad Hakim Khalili [a], Vishal Panchal [b], Alexander Dulebo [b], Sara Hawi [a], Rujing Zhang [c], Sandra Wilson [c], Eleftheria Dossi [d], Saurav Goel [e,f*], Susan A. Impey [a], and Adrianus Indrat Aria [a*]***

[a] Surface Engineering and Precision Centre, School of Aerospace, Transport and Manufacturing, Cranfield University, Cranfield MK43 0AL, U.K.

[b] Bruker UK Ltd, Banner Lane, Coventry CV4 9GH, U.K.

[c] Sophion Bioscience A/S, Baltorpvej 154, 2750 Ballerup, Denmark

[d] Centre for Defence Chemistry, Cranfield University, Shrivenham, Swindon SN6 8LA, U.K.

[e] London South Bank University, 103 Borough Road, London SE1 0AA, U.K.

[f] University of Petroleum and Energy Studies, Dehradun 248007, India

*** Corresponding author: a.i.aria@cranfield.ac.uk; ORCID: 0000-0002-6305-3906**

*** Corresponding author: goels@lsbu.ac.uk; ORCID: 0000-0002-8694-332X**

## 1. Schematic of printing setups for printing multilayer and monolithic hydrogels

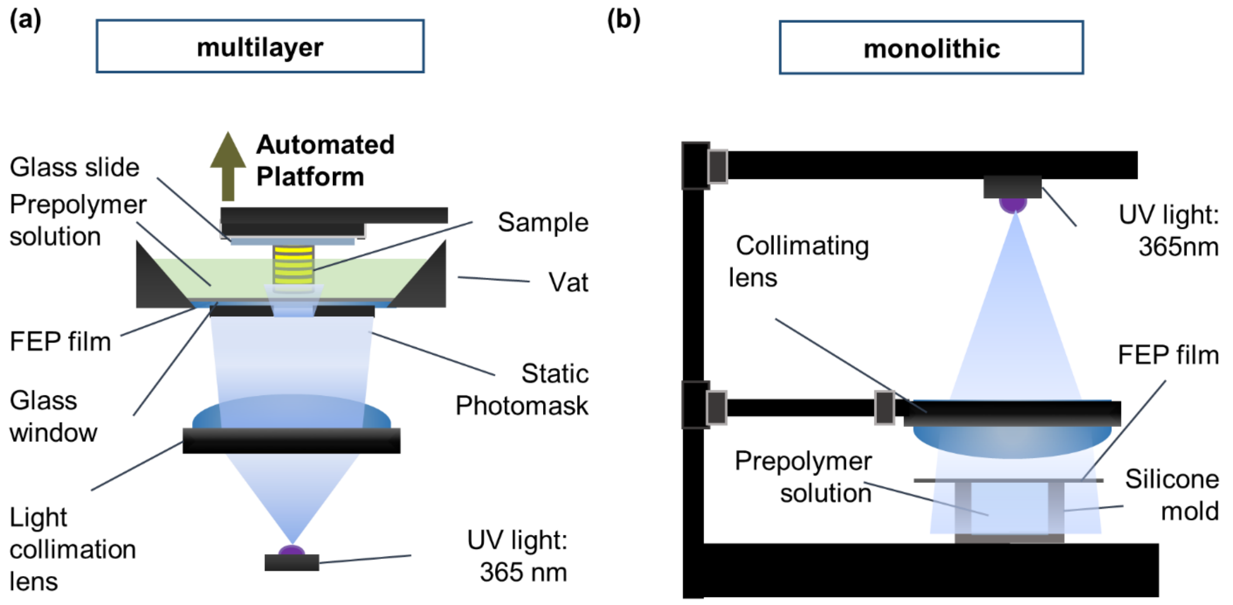


**Figure S1.** Schematic of (a) 3D printing projection lithography system for multilayer PEFDA hydrogels, (b) Bulk polymerisation setup for monolithic PEGDA hydrogels.

## 2. Analysis of force-distance (*P-h*) curves and contact point identification

The samples were initially analyzed by extracting force-distance (*P-h*) curves from NanoScope Analysis software (version 1.85) and JPK SPM Data Processing 8.0.13, and the elastic modulus (*E*) was estimated using both Hertz model and Oliver and Phar method by analyzing the extend (loading) and retract (unloading) curves separately. For using the Hertz model, to calculate the elastic modulus (*E*), a number of assumptions were made: (i) indentations are frictionless and for small strains, (ii) indentation depth is less than 1/3 of the probe radius, (iii) deformation is linearly elastic, (iv) for small fast indentations the viscoelasticity can be negligible.[1,2]

JPK SPM software was used for batch processing of the (*P-h*) curves by following the steps: (i) smoothen the data using the Gaussian function, (ii) subtract the baseline offset by fitting range of 75% to 95%, (iii) correct for any tilt, (iv) identify contact point by adjusting the x-offset where the contact point is assumed to be the first point at which the recorded force is in a positive value, (v) Hertz model for spherical indenter was used to the extend curve.[2,3]

$$P = \frac{E}{1-\vartheta^2}\left[\frac{a^2 + R_S^2}{2}\ln\frac{R_S + a}{R_S - a} - aR_s\right] \qquad \text{(S1)}$$

$$\delta = \frac{a}{2}\ln\frac{R_S + a}{R_S - a} \qquad \text{(S2)}$$

($P$) is the force, ($a$) is the radius of contact circle, ($\vartheta$) material Poisson's ratio, ($E$) is the elastic modulus of the material for a spherical tip with a radius ($R_s$) (eq 1).

The indentation depth ($\delta$) or the tip sample separation is measured from the identified contact point to the maximum displacement. The PEGDA Poisson's ratio is assumed to be 0.5.[4]

Analysis of (*P-h*) curves of AFM has always been challenging especially in locating the contact point to calculate the indentation depth which is a key parameter to measure *E*.[2,3,5] For hard samples, it is a straightforward step where it can be easily determined by using the extend curve and looking at the clear transition point. However, for soft samples, deformation due to long range forces happens before tip-sample contact, making the contact transition difficult to define and to differentiate between the cantilever movement and sample indentation depth. This can in turn can lead to large errors and deriving over/underestimated *E* for the sample.[6] In this study, we used a simple procedure where we defined the contact point as a point at which the recorded force crosses the y-axis for the first time. It should be noted that a few steps must be followed before identifying the contact point which is explained in the Methods and Materials section. Such an approach, even though not ideal, if used to analyze all (*P-h*) curves consistently can provide relatively good comparative results to understand the variation in the mechanical properties as a function of the build (printed) direction.

In Oliver and Pharr method the projected contact area of the indenter is determined based on the indentation depth. For fitting the (*p-h*) curves an external MATLAB script is used to fit the model to the retract curve where *E* is calculated following the retraction of the tip from the sample.[2] The step-by-step procedure can be found in the nature protocol where JPK software and the MATLAB script are used to extract the *E*.[2]

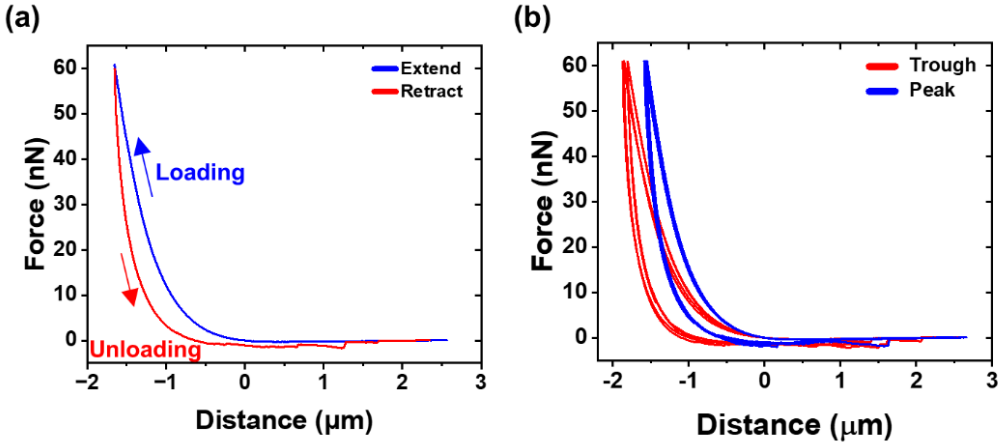


**Figure S2.** (a) A representative P-h curve of a multilayer PEGDA sample, indicating the extend (loading-blue color) and the retract (unloading-red color) sections of the curve. (b) (*P*-*h*) curves at different points across the peak and trough areas on the multilayer sample.

### 3. Indentation maps with location of every indent within a map of 90 x 90 µm

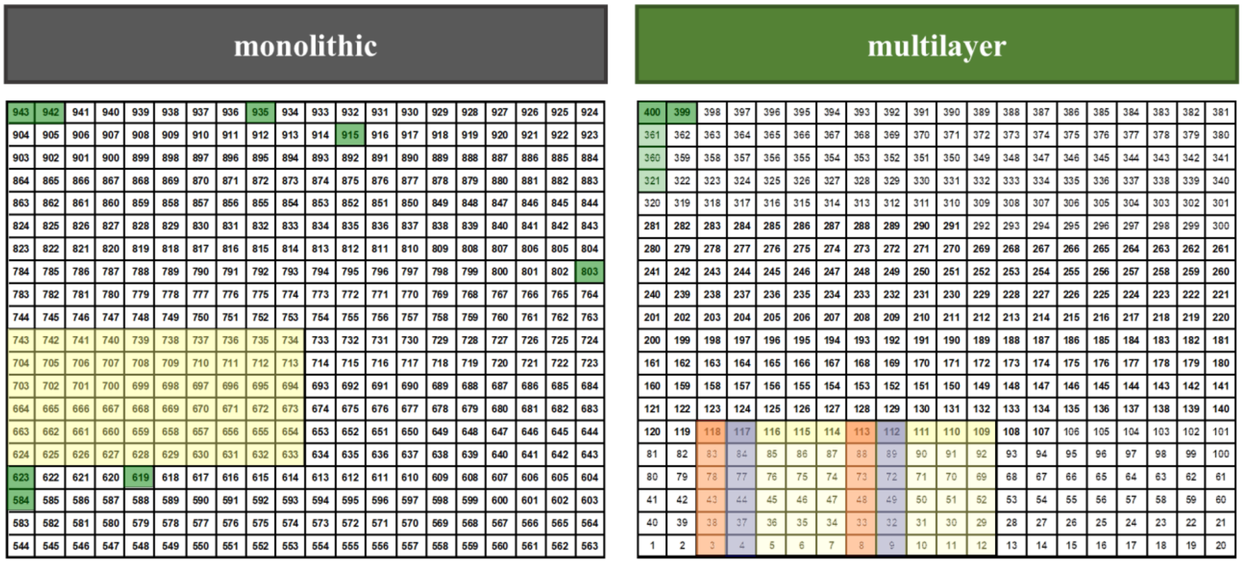


**Figure S3.** The indentation map with location of each indent within the map of 90 x 90 µm. The green indents are failed measurements omitted from the map. The red and blue colours represents indents on troughs and peaks, respectively. The highlighted area were analyzed in Figure 2.

## 4. Comparison of elastic modulus maps based on two different fitting ranges (Hertz model) and a fitting based on Oliver and Phar method

It is a common problem with soft materials to establish a contact point and to fit the unloading slope in the (P-h) curve (Figure S2a). To combat this issue, two different distance ranges, from 0.0 to 1.5 µm and from 0.5 to 2.0 µm, were chosen to fit the data for the slope to obtain *E* and a comparison of these results are shown in Figure S4a and b. These results showed that even though the magnitude of *E* increased when the higher range was used, the overall pattern and trend did not change. Therefore, it can be suggested that the results are comparable and point to a distinct and clear phenomenon concerning the change in *E* in a single layer of the multilayer sample. The lower fitting range was used for the remaining measurements. In terms of the difference between Hertz model (Figure S4a and b) and Oliver and Phar method (Figure S4c) it can be seen that the results are reporting a similar pattern; however, the magnitude of *E* has increased by 32% and 92% for both monolithic and multilayer samples, respectively when Oliver and Phar method was used. To measure *E* using the Oliver and Phar method, the approach involves identifying a suitable multiplier that can be utilized in the script to determine the correct contact point from the differential comparison between the first point and the subsequent points on the extension curve. This process involves examining each individual curve to ensure that the fitting is correct and the contact point has been accurately identified. However, this task is tedious and requires significant data processing time. As a result, we chose to use the Hertz model as the primary model for the study based on the assumptions outlined in the supporting information.

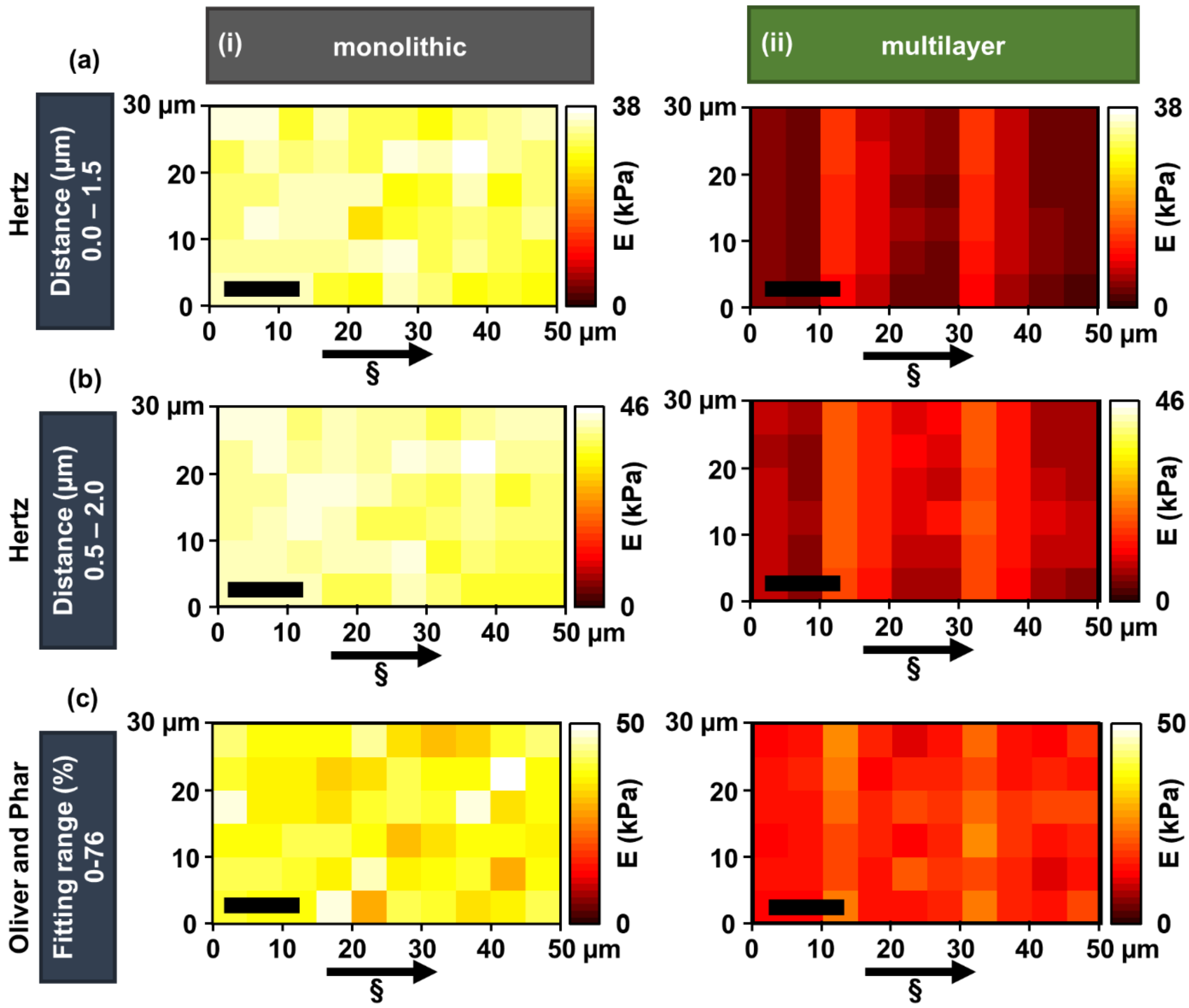


Figure S4. *E* map showing variation in modulus in both (i) monolithic, and (ii) multilayer PEGDA hydrogel samples based on two fitting ranges using Hertz model of (a) 0.0-1.5 µm, and (b) 0.5-2.0 µm. (c) *E* map using Oliver and Phar method. Scale bars in all graphs are approximately 10 µm.

## 5. Locations of indentation at 3 different heights on the multilayer hydrogel

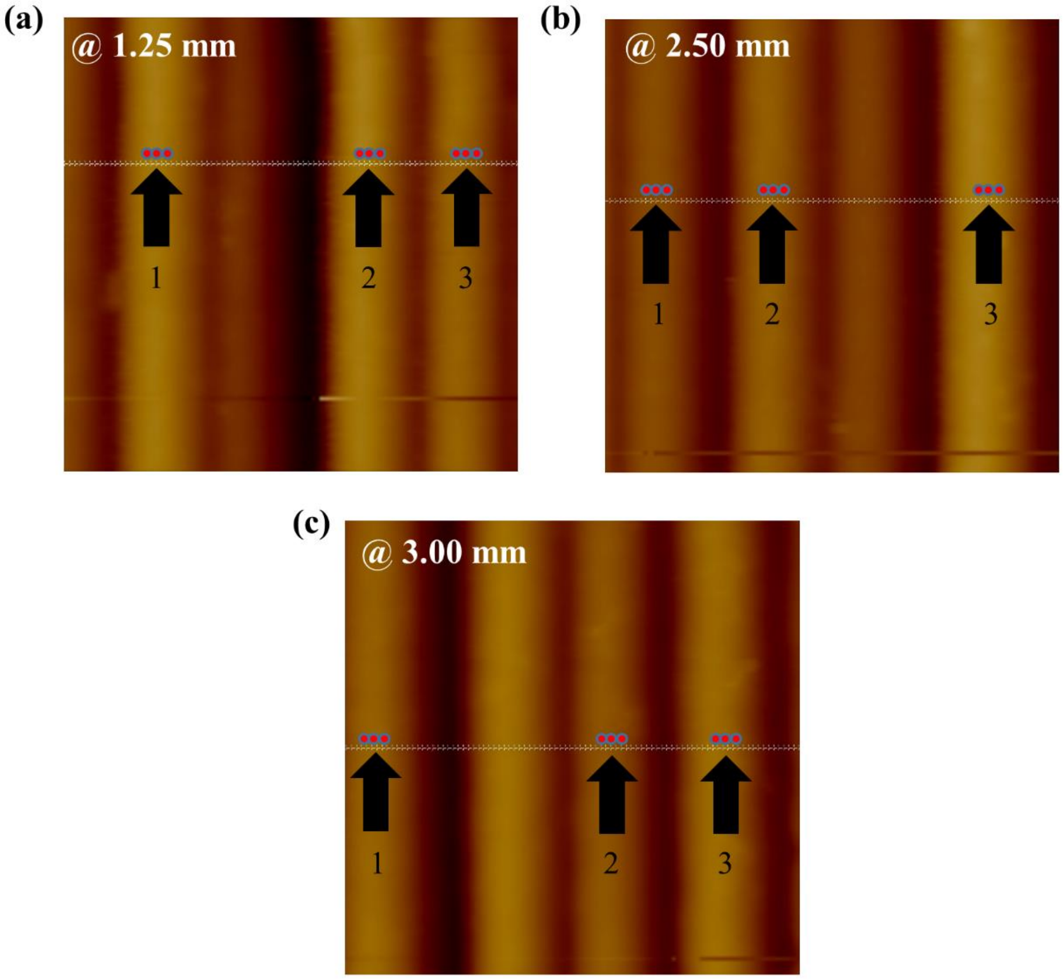


**Figure S5.** Locations of indentations at different heights on the multilayer hydrogel sample. The white horizontal line in each image is the indentation line (90 indents with 1 μm spacing) into the sample across the printed layers. The blue circles with red dots where the *E* was measured and the average *E* was based on.

## 6. Explanations of effect of surface topography on *E* measurements

In this study, two important arguments are addressed about the outer surface topography of the sample and the contact point. First, looking at Figure 2aii, it could be argued that the peak to valley (trough) depth in the multilayer sample is higher and therefore, the measured $E$ map may be influenced by how contact is established. In the Hertz model, eq S1 and S2, an ideal contact is assumed when the AFM tip is normal to the material's surface and the contact area follows the shape of the tip. Figure S6 postulates scenarios of the type of contact between the AFM tip and the sample's surface with red arrows highlighting the first contact point between the two objects. Given the size of the AFM tip (radius of 3.46 μm) and the printed layer thickness (20 μm), Figure S6ai shows how the contact may appear at the two points if the tip was approaching the interface between two consecutive layers, the trough. Conversely, if the tip is away from the interface, the contact scenario may differ and may occur at a single point (Figure S6aii). Two additional scenarios may also occur for the peak area of the printed layer where the tip may land exactly normal to the surface, the ideal situation, or on the side of the tip where the tip may slip on the surface rather than creating a well-established contact with the sample. Looking at Figure S5, where the exact position of the contact can be determined from the white dots on the apparent topography image, the scenarios mentioned above are plausible and cannot be ruled out.

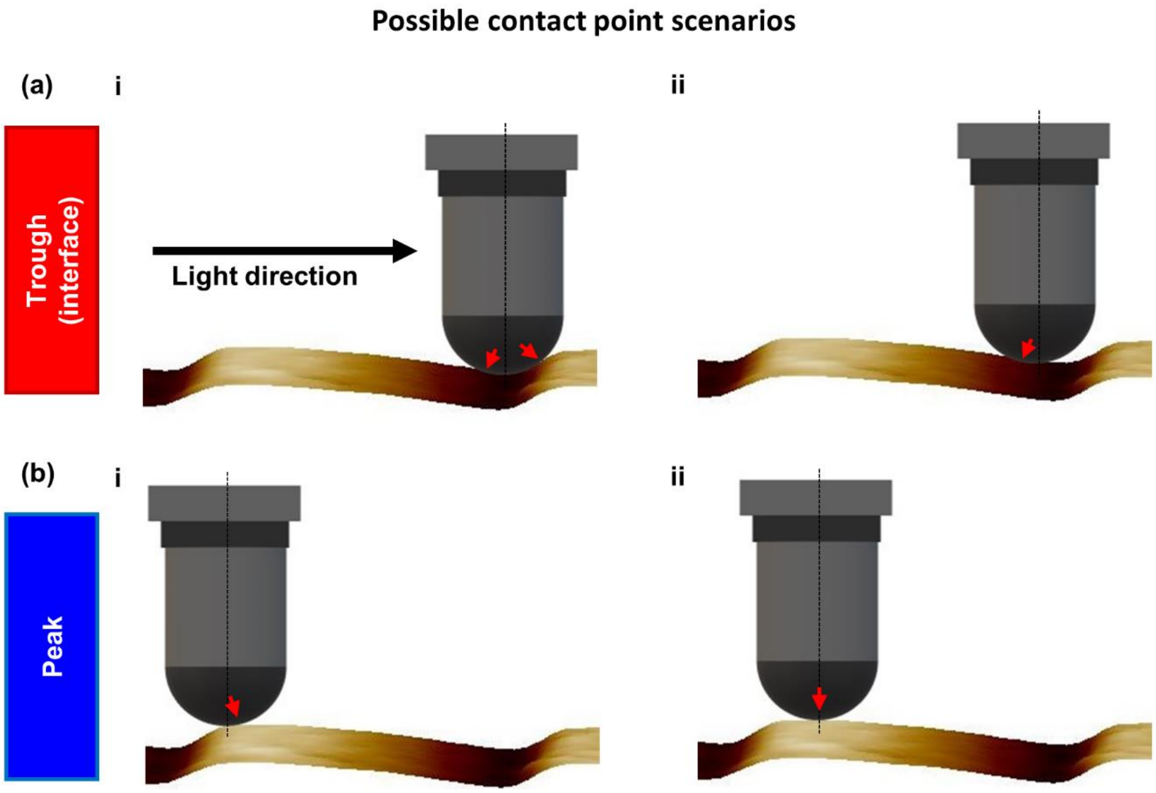


**Figure S6.** Schematic of different scenarios of contact between the AFM tip and the outer surface of the 3D multilayer PEGDA hydrogel at the interface. The red arrows demonstrate the contact points changing as the AFM tip moves from one area to another on the surface.

Secondly, since the surface topography is considered rough for the AFM measurements, it could be argued that there may be variations in terms of how much material is being indented beneath the surface which may influence the estimate of *E*. Since the indentation depth into the sample surface is well over 1 μm, the effect of surface topography on the measured *E* may be considered negligible.

## 7. Attempting to eliminate the outer surface topography

Attempting to eliminate the influence of the outer surface topography of the printed multilayer sample by reducing surface roughness, a cross-section of the multilayer sample was cut with 100 µm thick stainless steel blade in its hydrated state (Figure S7a). However, the surface topography and roughness increased with peak to valley (trough) difference reaching maximum of 5.3 and 6.7 µm in both multilayer and monolithic samples, respectively (Figure S7b), rendering it impossible to perform any sensible modulus measurements (Figure S7c). Another way to improve surface topography was to freeze and cryo-section the sample as it may improve the surface flatness.[7] However, such approach will completely change the hydrogel properties and its real-life condition and will lead to $E$ measurements that are effectively not representative of the real hydrated system.[8,9]

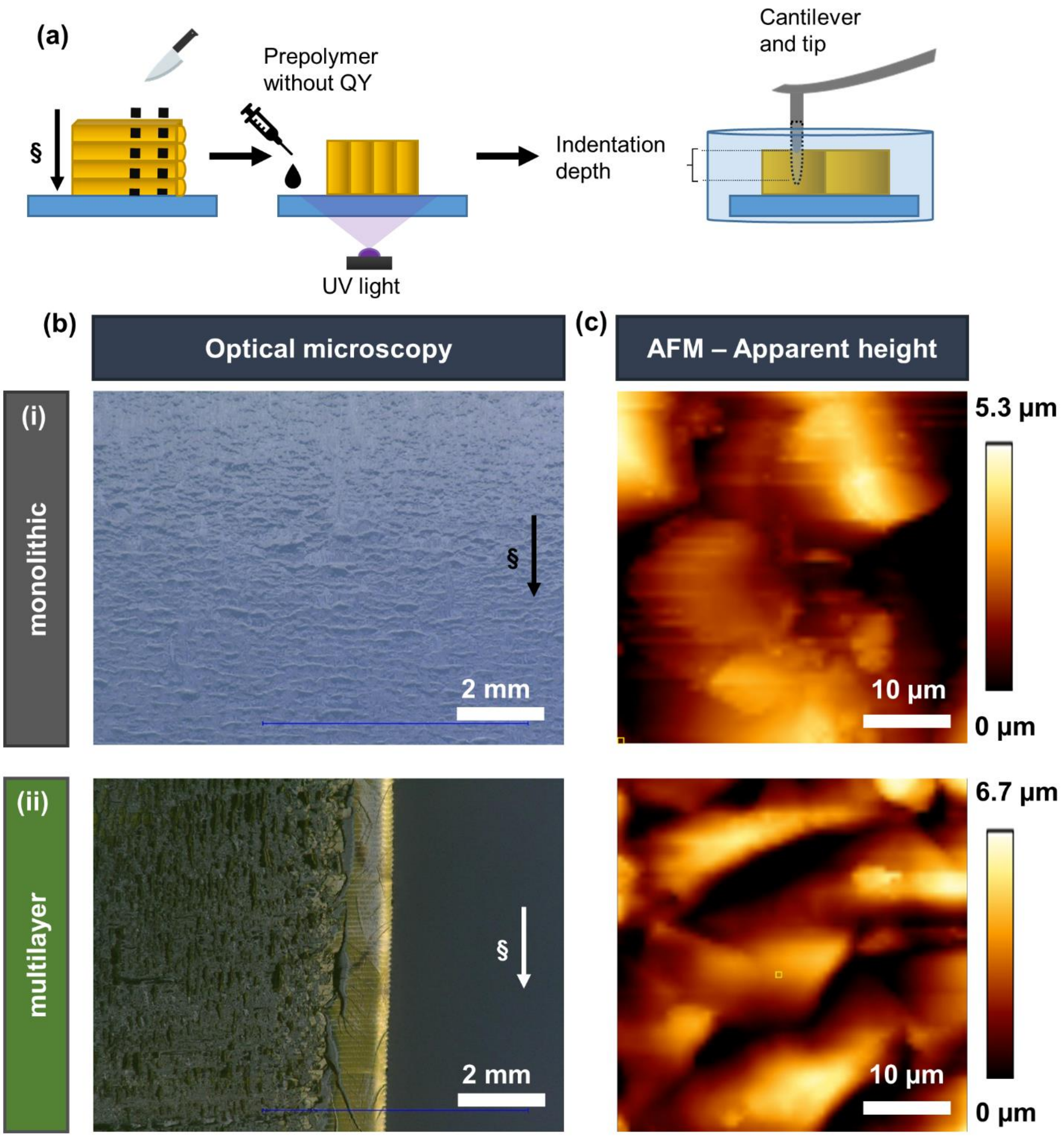


**Figure S7.** (a) Schematic of sample preparation for cross-section of 3D multilayer PEGDA hydrogel samples. (b) Optical microscope images of cross-sections, (c) AFM apparent height for (i) monolithic, and (ii) multilayer samples.

## 8. Measuring light absorbance for prepolymer solution with fixed QY concentration

A prepolymer solution containing 9 mg/mL QY, 5 mg/mL LAP, and 200 mg/mL PEGDA was prepared. The solution was poured onto a coverslip at the bottom of a vat, and the UV light intensity was adjusted to 20.51 mW/cm$^2$. After being exposed to UV light for 3 s, the excess solution was pipetted out from around the formed layer, and the surface of the layer was gently blotted with a paper towel. The thickness of the formed layer (known as cross-linking depth ($C_d$)) was measured and recorded as 46 ± 2 µm. The intensity of the UV light passing through the formed layer was measured and found to be 3.06 mW/cm$^2$. Using eq S3 the light absorbance was calculated:[10]

$$A = \log_{10}\left({}^{I_0}/_{I(L)}\right) \qquad \text{(S3)}$$

where ($I_0$) is the initial intensity at the bottom of the vat and $I(L)$ is the intensity of the light as it passes through the sample of thickness ($L$), the light absorbance was calculated to be 0.80.

A combination between eq S3 and Beer-lambert law eq S4 can be used to calculate the extinction coefficient ($\varepsilon$):[10]

$$A = \varepsilon L C \qquad \text{(S4)}$$

$$C_d = ({}^{1}/_{\varepsilon C}) \log_{10}\left({}^{I_0}/_{I(L)}\right) \qquad \text{(S5)}$$

where ($C$) is the concentration of the photoabsorber in the prepolymer solution and $L$ is the constant path light (which is equivalent to $C_d$ in this case). The extinction coefficient was calculated to be 5,474.6 M$^{-1}$ cm$^{-1}$.

## 9. Measuring cross-linking depth as light passes through a cross-linked single layer hydrogel with 20 µm layer thickness

Initially, single layer hydrogel with a thickness of 20 µm and a QY photoabsorber concentration of 9 mg/mL in the prepolymer solution was printed using an intensity of 20 mW/cm$^2$.The intensity of the UV light passing through the resulting cross-linked layer was measured to be 3.29 ± 0.1 mW/cm$^2$ (as depicted in Figure S9). Next, a glass slide was inserted into the printing vat and the UV light intensity was adjusted to 3.29 mW/cm$^2$. The same prepolymer solution used to print the 20 µm layer thickness was poured onto the glass slide and exposed to the adjusted UV light for 3 s (as shown in Figure S9). The resulting cross-linked layer was measured using a micrometer and found to have a thickness of 3 ± 2 µm. This suggests that only the top 3 µm of each layer in the multilayer sample were double-exposed (as illustrated in Figure S9), which is consistent with our previous findings and supports the formation of a highly cross-linked planar sub-layer.[11]

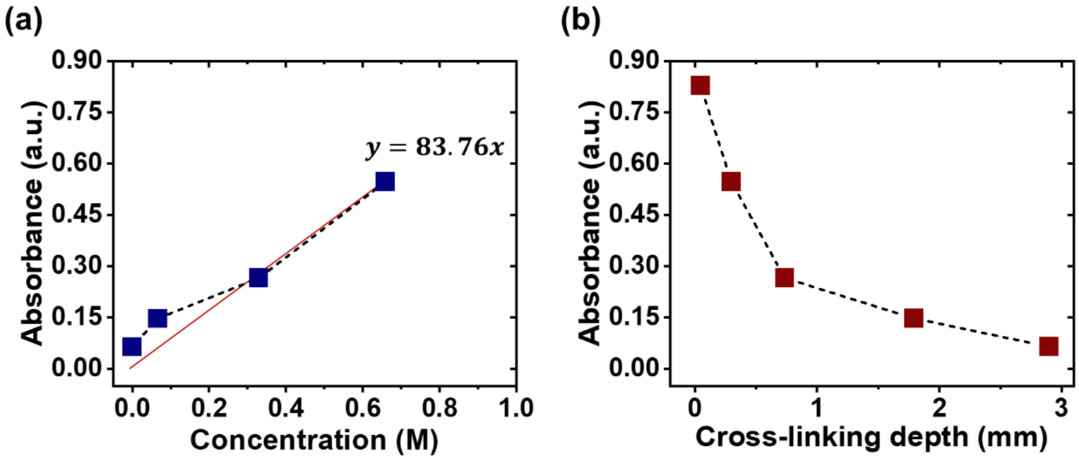


**Figure S8.** (a) Calibration curve of absorbance versus concentration at a fixed path length. (b) Absorbance versus cross-linking depth at different QY concentrations for a multilayer PEGDA hydrogel samples.

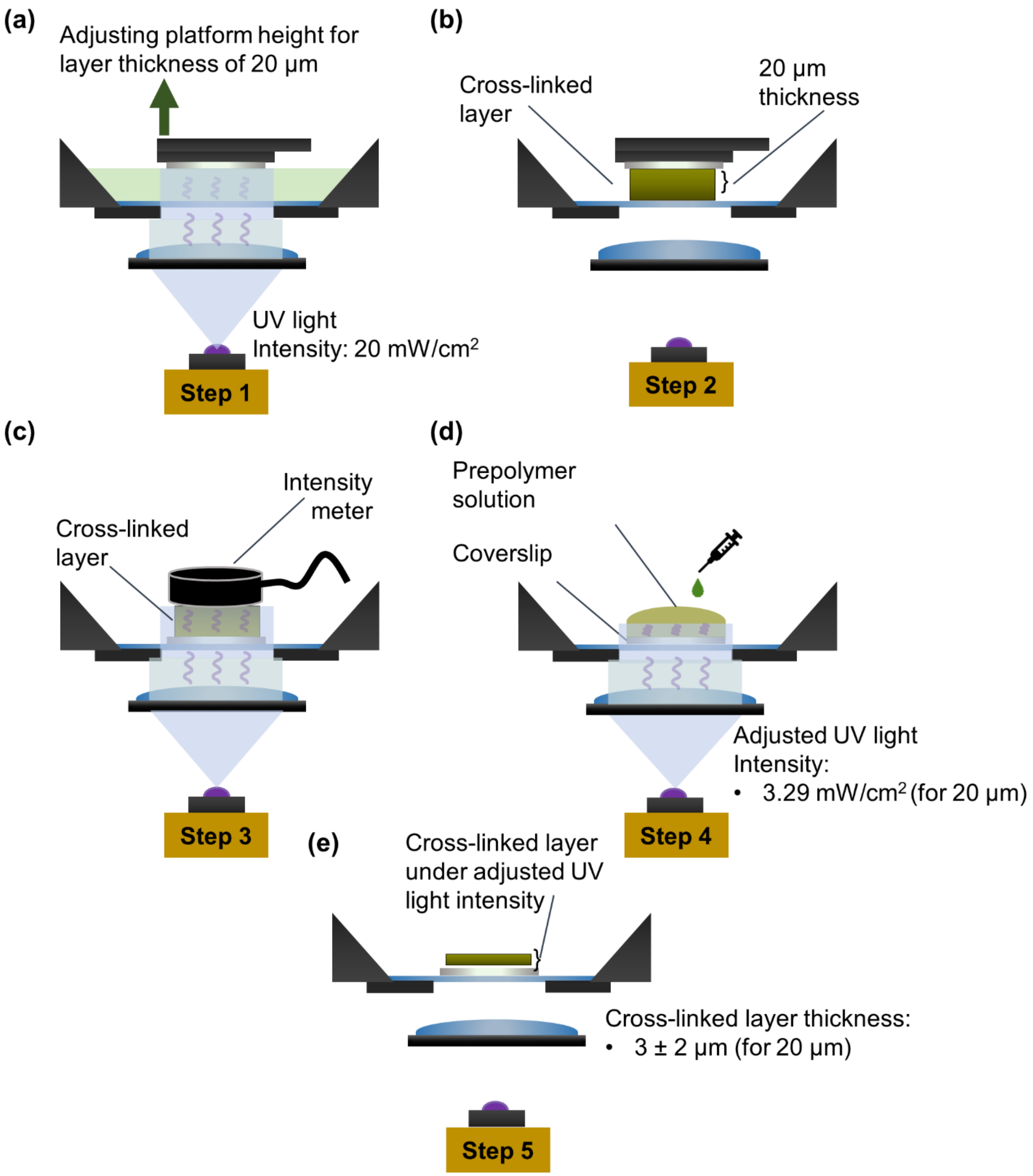


**Figure S9.** Schematic representation procedure for measuring the double exposed section in a 3D multilayer PEGDA hydrogel samples with layer thickness of 20 µm.

Based on our measurements, it appears that the intensity of the UV light passing through the prepolymer solution is insufficient to penetrate and double expose the entire previously formed

layer (as shown in Figure S10). The QY photoabsorber present in the prepolymer solution significantly absorbs light as it propagates through the solution during photo-cross-linking. This causes an exponential light intensity attenuation,[10,12] resulting in only a small portion of the top section of the previously formed layer near the interface being double exposed.

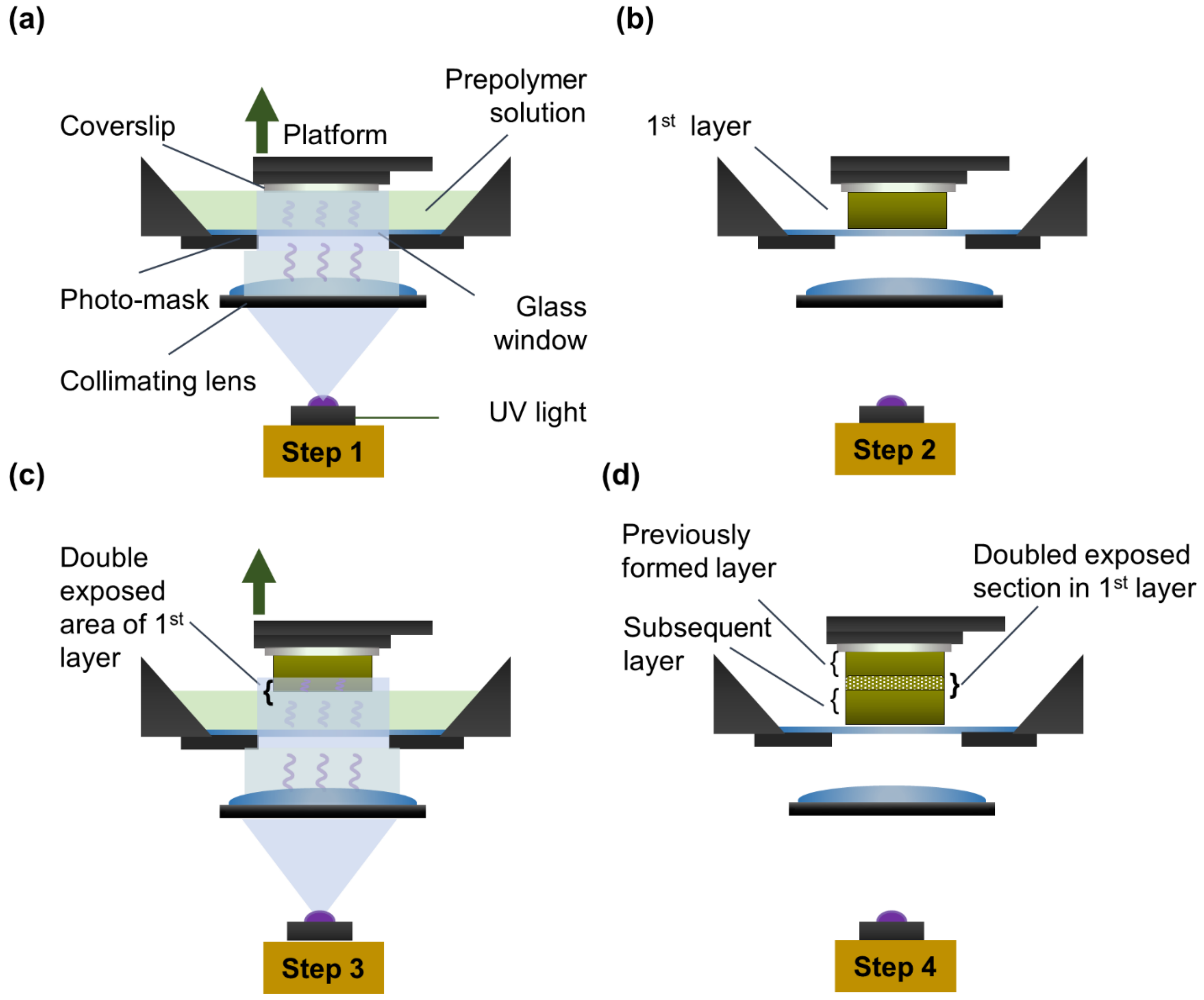


**Figure S10.** Schematic representation of the projection lithography system depicting the layer formation process (a-c) and (d) area within the surface of the previously formed layer which is potentially double exposed to UV light just below the interlayer interface.

## 10. Optical microscopic images of outer surface topography of multilayer hydrogels

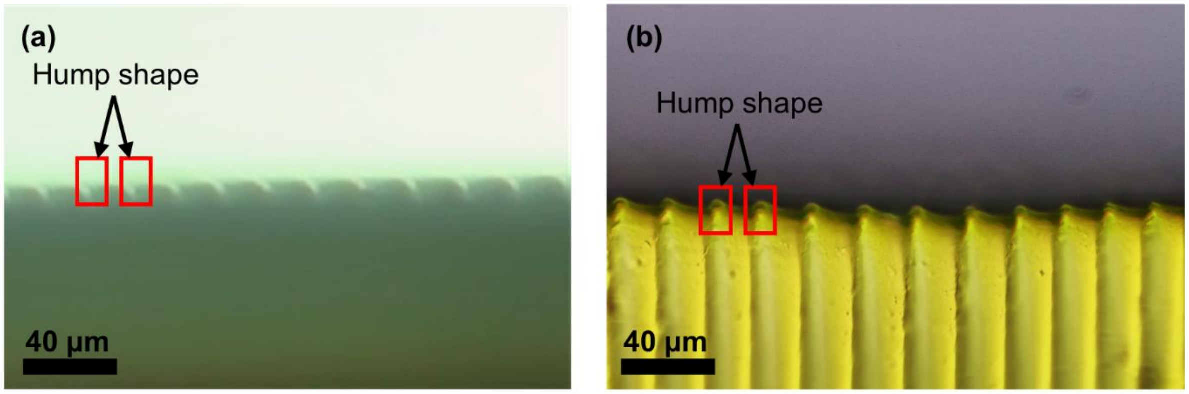


**Figure S11.** Optical microscopic image of the printed multilayer hydrogel sample outer surface topography of a (a) cylindrical, (b) cuboid shape samples.

## 11. Alternative options to LAP photoinitiator

There is a constant search for alternatives to metal-based photoinitiators in order to examine whether they can replace LAP and lead to a more homogeneous structure. If Irgacure 2959 was soluble in water instead of methanol, it could have been a viable option for tissue engineering studies.[1]> However, a more suitable photoinitiator with stable radicals such as TEMPO could be used to achieve well-controlled free-radical cross-linking. TEMPO's stable free-radical property is due to its steric bulk, which impedes the reaction of other free radicals, providing control over polymerization kinetics.[14] While TEMPO has been shown to be cytotoxic and unsuitable for tissue engineering, alternative options could be explored to examine if they may result in more homogenous structures.

### Reference


(1) Lin, D. C.; Horkay, F. Nanomechanics of Polymer Gels and Biological Tissues: A Critical Review of Analytical Approaches in the Hertzian Regime and Beyond. *Soft Matter* **2008**, *4*, 669–682. https://doi.org/10.1039/b714637j.

(2) Norman, M. D. A.; Ferreira, S. A.; Jowett, G. M.; Bozec, L.; Gentleman, E. Measuring the Elastic Modulus of Soft Culture Surfaces and Three-Dimensional Hydrogels Using Atomic Force Microscopy. *Nat. Protoc.* **2021**, *16*, 2418–2449. https://doi.org/10.1038/s41596-021-00495-4.

(3) Kontomaris, S. V.; Stylianou, A.; Malamou, A. Atomic Force Microscopy Nanoindentation Method on Collagen Fibrils. *Materials (Basel).* **2022**, *15*, 1–20. https://doi.org/10.3390/ma15072477.

(4) Gäbler, S.; Stampfl, J.; Koch, T.; Seidler, S.; Schüller, G.; Redl, H.; Juras, V.; Trattnig, S.;

Weidisch, R. Determination of the Viscoelastic Properties of Hydrogels Based on Polyethylene Glycol Diacrylate (PEG-DA) and Human Articular Cartilage. *Int. J. Mater. Eng. Innov.* **2009**, *1*, 1–20. https://doi.org/10.1504/IJMATEI.2009.024024.

(5) Simič, R.; Mandal, J.; Zhang, K.; Spencer, N. D. Oxygen Inhibition of Free-Radical Polymerization Is the Dominant Mechanism behind the "Mold Effect" on Hydrogels. *Soft Matter* **2021**, *17*, 6394–6403. https://doi.org/10.1039/d1sm00395j.

(6) Kilpatrick, J. I.; Revenko, I.; Rodriguez, B. J. Nanomechanics of Cells and Biomaterials Studied by Atomic Force Microscopy. *Adv. Healthc. Mater.* **2015**, *4*, 2456–2474. https://doi.org/10.1002/adhm.201500229.

(7) Gojzewski, H.; Guo, Z.; Grzelachowska, W.; Ridwan, M. G.; Hempenius, M. A.; Grijpma, D. W.; Vancso, G. J. Layer-by-Layer Printing of Photopolymers in 3D: How Weak Is the Interface? *ACS Appl. Mater. Interfaces* **2020**, *12*, 8908–8914. https://doi.org/10.1021/acsami.9b22272.

(8) Granke, M.; Does, M. D.; Nyman, J. S. The Role of Water Compartments in the Material Properties of Cortical Bone. *Calcif. Tissue Int.* **2015**, *97*, 292–307. https://doi.org/10.1007/s00223-015-9977-5.

(9) Oyen, M. L. Nanoindentation of Hydrated Materials and Tissues. *Curr. Opin. Solid State Mater. Sci.* **2015**, *19*, 317–323. https://doi.org/10.1016/j.cossms.2015.03.001.

(10) Benjamin, A. D.; Abbasi, R.; Owens, M.; Olsen, R. J.; Walsh, D. J.; Lefevre, T. B.; Wilking, J. N. Light-Based 3D Printing of Hydrogels with High-Resolution Channels. *Biomed. Phys. Eng. Express* **2019**, *5*, 1–10. https://doi.org/10.1088/2057-1976/aad667.

(11) Hakim Khalili, M.; Afsar, A.; Zhang, R.; Wilson, S.; Dossi, E.; Goel, S.; Impey, S. A.; Aria, A. I. Thermal Response of Multi-Layer UV Crosslinked PEGDA Hydrogels. *Polym. Degrad. Stab.* **2022**, *195*, 1–11. https://doi.org/10.1016/j.polymdegradstab.2021.109805.

(12) Otuka, A. J. G.; Tomazio, B.; Paula, K. T.; Mendonça, C. R. Two-Photon Polymerization: Functionalized Microstructures, Micro-Resonators, and Bio-Scaffolds. *Polymers (Basel).* **2021**, *13*, 1–30. https://doi.org/10.3390/polym13121994.

(13) Choi, J. R.; Yong, K. W.; Choi, J. Y.; Cowie, A. C. Recent Advances in Photo-Crosslinkable Hydrogels for Biomedical Applications. *Biotechniques* **2019**, *66*, 40–53. https://doi.org/10.2144/btn-2018-0083.

(14) Yu, C.; Schimelman, J.; Wang, P.; Miller, K. L.; Ma, X.; You, S.; Guan, J.; Sun, B.; Zhu, W.; Chen, S. Photopolymerizable Biomaterials and Light-Based 3D Printing Strategies for Biomedical Applications. *Chem. Rev.* **2020**, *120*, 10695–10743. https://doi.org/10.1021/acs.chemrev.9b00810.
